# Aristotle and adding an evolutionary perspective to models of plant architecture in changing environments

**DOI:** 10.3389/fpls.2013.00284

**Published:** 2013-07-31

**Authors:** Michael Renton

**Affiliations:** School of Plant Biology, The University of Western AustraliaPerth, WA, Australia

Why do plants grow the way that they do? According to Aristotle, there are four kinds of causes, or four fundamentally different ways of answering “why” questions such as this (Aristotle, 1984; Falcon, 2012). In reductionist science, answers to “why” questions typically relate to one of the first three of Aristotle's causes, regarding changes in substances (material cause), in form (formal cause) and in the effects of external influences (efficient cause). This is reflected in much functional-structural pant modeling (FSPM), where “structural” aspects of plant architecture are clearly concerned with formal causes and internal “functional” aspects, such as hormones and transported nutrient are clearly concerned with material causes (Sievänen et al., 2000; Prusinkiewicz, 2004; Yan et al., 2004; Godin and Sinoquet, 2005; Fourcaud et al., 2008; Hanan and Prusinkiewicz, 2008; Vos et al., 2010). The environmental aspects, such as light, soil water and nutrients, pests and pathogens that are also often included in such FSPM and interact with both function and structure are clearly concerned with efficient causes. However, Aristotle's fourth kind of cause, final cause, seems to be less considered in reductionist science in general, and in FSPM in particular.

Final causes concern the aim or purpose being served by the object of interest, a plant in our case. In other words, discussion of final causes concerns answering the question of why a plant grows the way it does by reference to the purpose of that growth. Such answers could take the form of “The plant is growing like that because it is trying to maximize its light interception,” for example. In science, such a response may lead to accusations of anthropomorphism, which can be defined as the attribution of human qualities to things other than humans, with a connotation that such attribution is erroneous and problematic (Horowitz, 2007). If Pavlov (1927) wrote that animals should be “studied as purely physiological facts, without any need to resort to fantastic speculations as to the existence of any possible subjective state in the animal which may be conjectured on analogy with ourselves,” then it would seem an even greater sin to explain the behavior of plants as “purposeful,” or in terms of what they are trying to achieve with that behavior? However, evolutionary theory provides a clear rationale for the value of explanations of behavior in terms of the purpose of that behavior, as long as it can be seen as having an evolutionary advantage, and thus having been selected for by evolutionary processes. So we can rephrase our “final cause” response more carefully, “The plant is growing like that because that is an ecological strategy that has evolved over time due to the fact that it tends to maximize the plant's light interception.” But how can we know whether a growth strategy has indeed evolved over time to maximize light interception (or any other function that contributes to evolutionary success)?

The dynamic structural development of a plant can be seen as a strategy for exploiting the limited resources available within its environment, such as light, soil water and nutrients, and we would expect that evolution would lead to efficient growth strategies that reduce resource costs while maximizing resource acquisition. No one growth strategy will be optimal in all environments; which strategies of structural development are most effective will depend on how the resources on which the plant depends are distributed through both time and space. The relative advantage of a plant's growth strategies will also depend on how its architecture influences factors such as dispersal of seeds and pollen, the impacts of herbivoury and drought stress, the efficiency of water transport, biomechanical support, and resistance to wind, along with how much it costs to produce and maintain the structures that comprise its architecture (Küppers, 1989; Gartner, 1995). Therefore, if we are to shed light on Aristotle's final cause and start to understand why plants have evolved different strategies of structural development, we need to understand the various costs and benefits of different growth strategies in different environments (Farnsworth and Niklas, 1995; Lynch, 1995).

There is a long history of modeling plants in order to investigate the costs and benefits of different structural growth strategies (e.g., Shinozaki et al., 1964; Honda and Fisher, 1979; Johnson and Thornley, 1987; Niklas, 1999; West et al., 1999; Takenaka et al., 2001; Falster and Westoby, 2003; King et al., 2003). However, many potentially important aspects of plant growth and function have not been represented in these models, largely due to computational constraints and limitations in modeling technology. As simplifications of reality, no model can possibly include all aspects of reality. Nonetheless, recent years have seen the development of a new generation of plant models that include more of these previously neglected aspects, such as the explicit topology and spatial geometry of the plant structure; the way that the plant architecture develops dynamically over time by changes in existing components and the addition of new ones; the feedbacks between plant structure, function, and environment that also change with time as the plant grows and the environment changes; the way that the distribution of resources within a plant's environment varies with time and space; and competition between individuals within plant populations and communities. It is this “new generation” of models that are often known as functional-structural plant models (FSPMs) or “virtual plants” (Sievänen et al., 2000; Prusinkiewicz, 2004; Yan et al., 2004; Godin and Sinoquet, 2005; Fourcaud et al., 2008; Hanan and Prusinkiewicz, 2008; Vos et al., 2010).

The fact that FSPMs represent a large number of potentially-important interacting processes in a dynamic way and at a high degree of detail would seem to make them a perfect tool for investigating the costs and benefits of different structural growth strategies, and thus providing insight into the final cause of plant growth strategies. Indeed many models that could be termed FSPMs have been employed to investigate the relative advantages of varying below- and above-ground structural growth strategies (e.g., Pearcy and Yang, 1996; Colasanti and Hunt, 1997; Dunbabin et al., 2003; Pearcy et al., 2005; Sterck et al., 2005; Clark and Bullock, 2007; Pagès, 2011). However, the strength of FSPMs, their dynamic realism, is also their weakness, because it makes them relatively complex and computationally demanding. It can take a relatively long time to run even a single FSPM growth simulation, and an FSPM typically contains a large number of growth-strategy-defining parameters, meaning that to run simulations for all combinations of all values of all parameters of interest becomes a major computational challenge. One approach is to use a relatively complex and realistic FSPM but only attempt to evaluate a limited subset of all possible strategies (e.g., Dunbabin et al., 2003; Pagès, 2011), and the other is to use a simpler FSPM but explore a more comprehensive set of strategies (e.g., Niklas, 1994, 1999). However, probably neither of these would really satisfy Aristotle in his search for a final cause of real plant growth; for that we need a thorough and comprehensive search through a wide range of growth strategies with a model that is flexible and detailed enough to capture the most important aspects of real plant growth.

**Figure 1:**
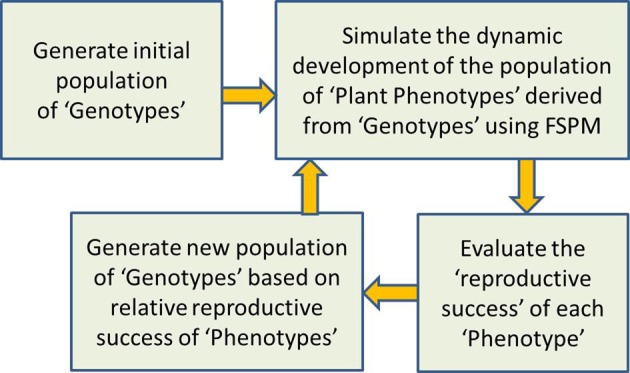
**General schema of how an evolutionary algorithm can be combined with a FSPM to investigate the final cause or “evolutionary purpose” of plant growth**. The FSPM to be used would have a number of parameters that define its growth strategy, and it is assumed that these parameters represent genetic information that can change with evolution. First an initial “population” of “genotypes” is generated, with each “genotype” consisting of a different set of values for all growth strategy parameters. In step two, the “phenotypic” realization of each “genotype” is simulated with runs of the FSPM, each one corresponding to a set of growth strategy parameters. In step three, the relative reproductive success of each phenotype is determined; this could be based on the final size of the plant for example, with larger plants assumed to produce more seed and pollen and thus be more likely to contribute genes to following generations, all else being equal. In step four, these measures of relative reproductive success are used to generate a new population of genotypes; for example, the genotype of each new seed would be based on the genotype of one or two randomly selected “parent phenotypes,” with the chance of a simulated plant being chosen as a parent depending on its size. Step two is now applied to the new population of genotypes, resulting in a new population of phenotypes, and so the process continues until a specified number of generations have elapsed, or until some other criterion indicating sufficient evolution is satisfied.

A promising option for moving forward is to employ evolutionary optimization algorithms (Fogel, 1994; Ashlock, 2006). Such algorithms provide a computationally efficient means of exploring a wide range of possibilities in search of optimal solutions. In addition, marrying evolutionary algorithms with FSPMs would also appear to be a perfect way to explore the optimality of plant structures and growth strategies from an evolutionary perspective, in order to deepen our understanding of the relationships between evolution, ecosystems, individual plants, and genes (Prusinkiewicz, 2000). Earlier use of evolutionary algorithms with models of plant structure were aimed at evolving better above-ground plant forms based on aesthetic criteria (McCormack, 1993; Jacob, 1994; Traxler and Gervautz, 1996; McCormack, 2004); these representations of plant structure were relatively simple and abstract and contained little realistic representation of biological processes. In more recent times, more biologically-motivated questions of ecological theory and above-ground plant competition at the level of individual plants and plant populations have been tackled with a combination of structural plant models and evolutionary computation, but still at a relatively abstract level (Bornhofen and Lattaud, 2006, 2007, 2009; Kennedy, 2010; Bornhofen et al., 2011). These examples only highlight the huge potential for using sophisticated evolutionary computation with more detailed and realistic FSPMs. While the potential focus of such FSPMs is almost limitless (above-ground, below-ground, herbs, shrubs, trees…), the way that an evolutionary algorithm can be combined with a FSPM to investigate the final cause of plant growth can be explained in quite general terms (Figure 1).

In a recent study illustrating the potential of this approach, we explored below-ground plant structural optimality by linking an evolutionary optimization algorithm with a dynamic root growth FSPM (Renton et al., 2012; Renton and Poot, 2013, unpublished) in a Tool for Analysis of Root Structures Incorporating Evolution of Rooting Strategies (TARSIERS). This study extended on previous studies by including a relatively detailed representation of root structure and spatial and temporal variations in resource distributions, applied to a realistic case study situation—perennial plants growing on shallow soils in seasonally dry environments. The approach was able to simulate reasonable patterns of evolution of structural growth strategies that converged toward the specialized root system morphologies that have been observed in species restricted to these types of habitats, and which are likely to enhance access to water resources in cracks in the underlying rock (Poot and Lambers, 2003a,b, 2008; Poot et al., 2008, 2012). The study showed how adding an evolutionary perspective to FSPMs could provide insights into both evolutionary processes and the ecological costs and benefits of different plant growth strategies.

As computing technology and modeling methodologies continue to advance, the computational difficulties of applying comprehensive and realistic evolutionary algorithms to detailed and realistic models of plant structure and function will continue to be overcome. While the realm of possibilities will keep expanding, the challenge will continue to be to design plant models that are simple enough for evolutionary optimization to be computationally feasible, yet flexible enough to allow a range of structural development strategies to be explored and realistic enough to capture the essential characteristics of interest. Within current FSPMs, the representation of the interactions between functional processes and structural development can be relatively simple and empirical (Renton et al., 2005a,b, 2007) or more mechanistic, realistic, detailed, and thus complex (Allen et al., 2005; Costes et al., 2008; Lopez et al., 2008); it is likely that relatively simple approaches will be of most use for integration into evolutionary simulations in the foreseeable future, although the use of “super-computing” facilities could potentially allow evolutionary optimization to be applied to even very complex and detailed FSPMs. The approaches developed will give insights into both evolutionary processes and the ecological costs and benefits of different plant growth strategies. The strategies considered could include both fixed strategies, which do not depend on the environment encountered by an individual plant, and plastic strategies, that do adapt to the encountered environment. By showing how plant architectural strategies have evolved to meet the requirements of certain specific environments, they will also help understand and predict how these strategies are likely to function or adapt as environments change in the future. If, as Dobzhansky (1973) wrote, “nothing in biology makes sense except in the light of evolution,” then it is essential to add an evolutionary perspective to FSPM, which addresses Aristotle's fourth and final cause in addition to his first three causes addressed by the structural, functional, and environmental perspectives already commonly used in FSPM. This will help to provide a more complete answer to the question of why plants grow the way they do.
